# Endovascular Coil Embolization of a Ruptured Gastroduodenal Artery Aneurysm: A Case Report

**DOI:** 10.7759/cureus.111268

**Published:** 2026-06-21

**Authors:** David Shengelia, Mariam Mamaiashvili, Giorgi Kentchadze, Lasha Mukhigulashvili

**Affiliations:** 1 Radiology, American Hospital Tbilisi, Tbilisi, GEO; 2 Interventional Radiology, American Hospital Tbilisi, Tbilisi, GEO; 3 Vascular and Endovascular Surgery, American Hospital Tbilisi, Tbilisi, GEO; 4 Vascular Surgery, American Hospital Tbilisi, Tbilisi, GEO

**Keywords:** chronic pancreatitis, endovascular coil embolization, endovascular management, gastroduodenal artery aneurysm, interventional radiology

## Abstract

Gastroduodenal artery aneurysms (GDAAs) are among the rarest forms of visceral artery aneurysms. Despite their rarity, GDAAs carry a high risk of rupture associated with significant morbidity and mortality, making early diagnosis and prompt intervention essential. Endovascular embolization has increasingly emerged as a minimally invasive alternative to open surgical repair.

We present the case of a patient admitted with progressively worsening diffuse abdominal pain accompanied by one episode of black-colored vomiting. Contrast-enhanced computed tomography (CECT) revealed findings consistent with rupture of a GDAA. Due to the acute clinical presentation and risk of ongoing hemorrhage, the patient underwent emergency endovascular coil embolization.

The procedure was successfully performed by selective catheterization of the gastroduodenal artery and proceeded without complications, resulting in hemodynamic stabilization and avoidance of open surgical intervention. This case highlights the importance of rapid radiologic diagnosis and demonstrates that endovascular coil embolization is a safe and effective minimally invasive treatment option for ruptured GDAA.

## Introduction

Gastroduodenal artery (GDA) aneurysm (GDAA) is an uncommon but potentially life-threatening vascular abnormality, accounting for approximately 1.5% of all visceral artery aneurysms. Despite their rarity, they carry a high risk of rupture (reported up to 35%) and a mortality rate approaching 40% when rupture occurs [1], so they should be treated promptly regardless of their size. Chronic pancreatitis and atherosclerosis are among the most frequently reported etiological factors [2], while other causes include autoimmune disorders, abdominal trauma, and infections.

Clinical presentation is often nonspecific and may range from incidental asymptomatic findings to acute abdominal pain, gastrointestinal hemorrhage, gastric outlet obstruction, or hemobilia [3]. Because the GDA runs in close proximity to the pancreatic head and duodenum, chronic pancreatitis and peptic ulcer disease contribute to vascular wall injury through persistent inflammation, enzymatic degradation, and local tissue erosion, increasing the risk of aneurysm formation [2].

According to the Society for Vascular Surgery (SVS) [4] clinical practice guidelines on the management of visceral aneurysms, coil embolization is recommended as the preferred treatment modality for both intact and ruptured GDAAs [5]. We report a case of a 54-year-old male patient diagnosed with GDAA rupture, who was treated successfully with endovascular coil embolization. The successful outcome observed in our patient is consistent with these recommendations and further supports the role of endovascular therapy as a safe and effective first-line treatment option. 

## Case presentation

A 54-year-old male patient was admitted to the hospital with diffuse abdominal pain that had started one day prior and had progressively increased in intensity. He also reported a single episode of black-colored vomiting. There was no history of previous similar complaints or known chronic medical conditions.

On admission to the emergency department, abdominal ultrasound revealed a heterogeneous rounded lesion in the region of the liver hilum measuring approximately 49 × 50 mm. Initial laboratory investigations (Table 1) demonstrated leukocytosis (WBC 16.2 × 10⁹/L), elevated serum lipase levels (LIP, 120 U/L), mildly elevated C-reactive protein (CRP, 25.73 mg/L), and increased serum lactate levels (Lac, 2,4 mmol/L). Pre-procedural laboratory evaluation showed mild anemia and a mildly elevated platelet count. Despite these findings, no blood transfusion was required before or after the procedure. 

**Table 1:** Preoperative laboratory results. CRP, C-reactive protein; WBC, white blood count; LIP, lipase; Lac, lactate; HGB, hemoglobin; HCT, hematocrit; PLT, platelets

Test	Result	Unit	Normal range
CRP	25.73	mg/L	<5
WBC	16.2	10^9/L	4.00-9.00
LIP	120.1	U/L	13.00-60.00
Lac	2.4	mmol/L	0.9-1.7
HGB	11.7	g/dL	13.50-18.00
HCT	33.4	%	38.00-52.00
PLT	423.0	10^9/L	150.00-400.00

Based on these findings, further evaluation with contrast-enhanced computed tomography (CECT) was performed.

Computed tomography findings

Contrast-enhanced CT demonstrated a 3.4 × 3.5 cm aneurysm adjacent to the pancreatic head, with a patent lumen measuring up to 2.8 cm and containing intramural hematoma and peripheral calcifications. A surrounding 5 × 5.5 cm hemorrhagic collection was identified, causing compression and narrowing of the second and third portions of the duodenum, consistent with aneurysm rupture. The stomach was markedly distended and contained hyperdense material compatible with blood products, with similar hyperdense contents seen within the small bowel lumen. Additional findings included features of chronic pancreatitis, including diffuse pancreatic calcifications and dilatation of the main pancreatic duct (10-12 mm). The celiac trunk and superior mesenteric artery demonstrate normal origin and branching pattern. Extensive atherosclerotic changes of the abdominal aorta and iliac arteries were also present. These findings were most consistent with rupture of the GDA (Figures 1-2). 

**Figure 1:**
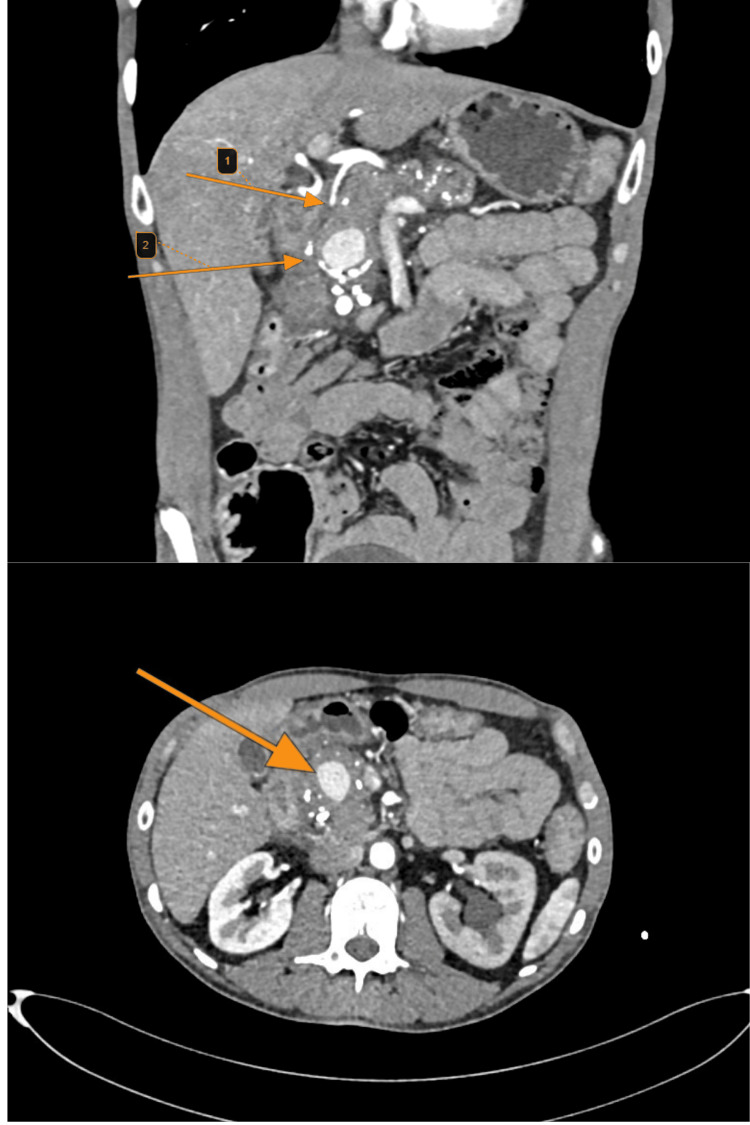
Preoperative coronal (above) and axial (below) contrast-enhanced computed tomography (CECT) of the abdomen. The upper image demonstrates a coronal section of the abdominal cavity. Arrow 1 (orange) indicates the gastroduodenal artery with normal contrast opacification. Arrow 2 (orange) highlights a large hyperdense vascular lesion in the region of the gastroduodenal artery with features suspicious for active contrast extravasation. Surrounding inflammatory changes and soft-tissue density are noted in the pancreaticoduodenal region. The lower image with the orange arrow demonstrates pooling of contrast media outside the margins of the GDAA. GDAA, gastroduodenal artery aneurysm

**Figure 2:**
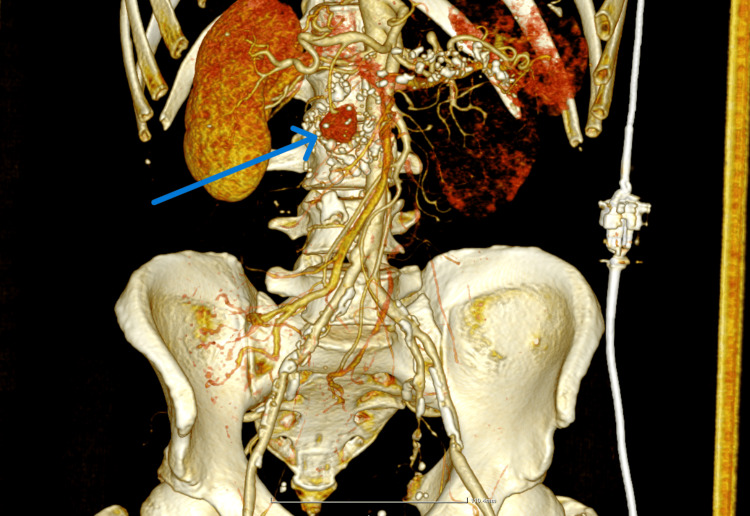
Three-dimensional computed tomography angiography. Volume-rendered reconstruction demonstrating a ruptured gastroduodenal artery aneurysm with active contrast extravasation (blue arrow).

Following multidisciplinary consultation with interventional radiology and vascular surgery teams, urgent endovascular treatment was indicated. 

Endovascular treatment

The procedure was performed under local anesthesia via ultrasound-guided brachial artery access using the Seldinger technique. A 5F introducer sheath was placed, and a guidewire was advanced into the aorta and subsequently into the celiac trunk. A diagnostic catheter was positioned in the GDA, and angiography confirmed the diagnosis of a GDAA (Figure 3). 

**Figure 3:**
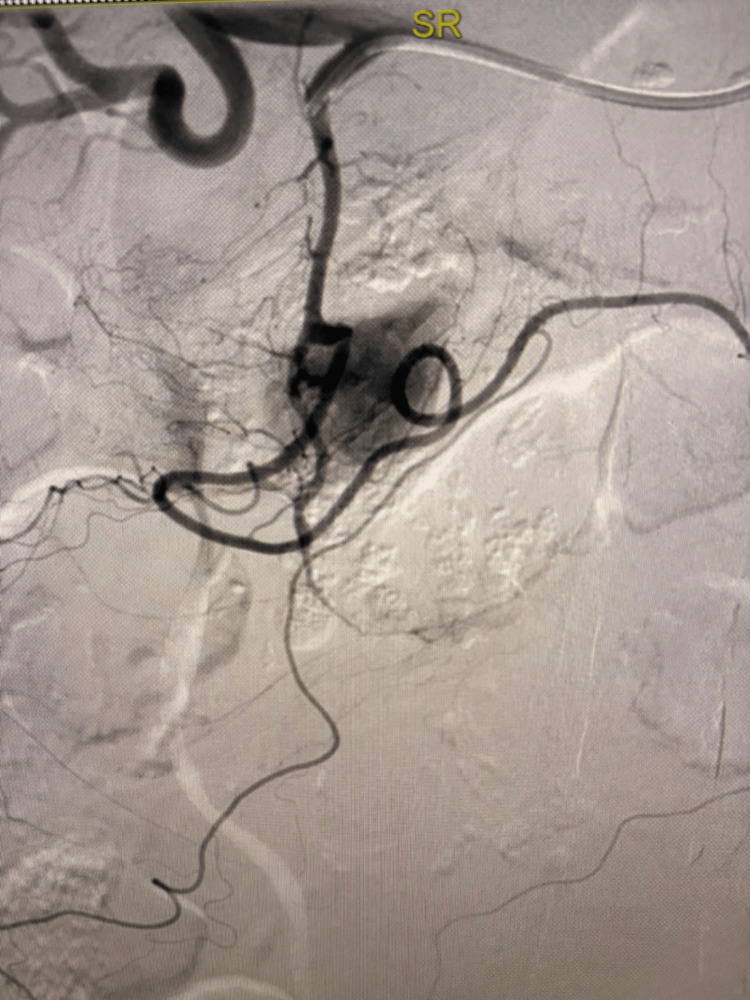
Digital subtraction angiography demonstrating rupture of a gastroduodenal artery aneurysm. Selective catheterization of the gastroduodenal artery revealed active contrast extravasation from the aneurysmal sac, consistent with aneurysm rupture and ongoing hemorrhage.

Brachial arterial access was chosen because it allows early patient mobilization and may improve postprocedural comfort compared with femoral access. Following femoral artery catheterization, patients often require a period of bed rest and limb immobilization, whereas brachial access permits earlier ambulation.

Selective catheterization of the affected vessel was achieved using a microcatheter system, followed by coil embolization of the GDA using micro-coils: four 5 mm × 3 cm coils, three 3 mm × 3 cm coils and one 5 mm × 2 cm coil. Final control angiography, including selective angiography of the superior mesenteric artery, demonstrated complete exclusion of the aneurysm with no further opacification of the lesion or the GDA (Figure 4). Hemostasis was achieved by manual compression, and no access-site complications, including hematoma, pseudoaneurysm or arterial thrombosis, were observed.

**Figure 4:**
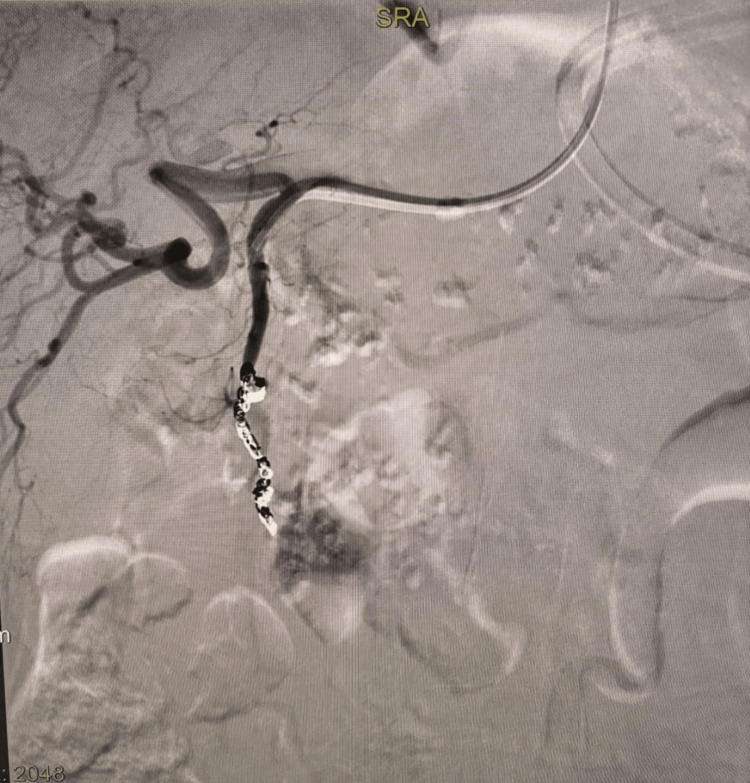
Post-embolization angiography following coil embolization of the ruptured gastroduodenal artery aneurysm. Completion angiography demonstrates successful occlusion of the gastroduodenal artery aneurysm after coil embolization, with no residual filling of the aneurysmal sac or evidence of contrast extravasation.

Outcome and follow-up

Post-procedural CT confirmed successful coil embolization with no contrast filling of the aneurysm sac (Figure 5).

**Figure 5:**
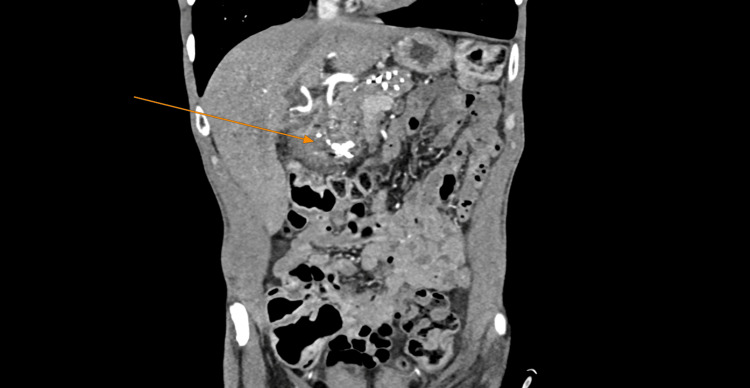
Post-procedural coronal computed tomography following endovascular coil embolization of a ruptured gastroduodenal artery aneurysm. Contrast-enhanced computed tomography (CECT) demonstrates embolization coils within the gastroduodenal artery at the site of the previously identified aneurysm (orange arrow). No evidence of residual aneurysm enhancement or active contrast extravasation is observed. Successful exclusion of the aneurysm was supported by persistent occlusion of both the afferent and efferent segments of the gastroduodenal artery, with no evidence of residual perfusion.

Post-procedural management included intravenous fentanyl for analgesia, intravenous pantoprazole for gastric protection and intravenous 0.9% sodium chloride for hydration. The patient's abdominal pain progressively improved and was markedly alleviated by the second post-procedural day. Thereafter, any mild residual pain was effectively controlled with nonsteroidal anti-inflammatory drugs (NSAIDs). At the time of discharge, the patient was pain-free, hemodynamically stable and had normal vital signs (blood pressure, 112/66 mmHg; heart rate, 73 beats/minute; respiratory rate, 18 breaths/minute; oxygen saturation, 99% on room air). The patient was discharged home on day 5 with instructions to use NSAIDs as needed for pain, together with pantoprazole for gastric protection.

## Discussion

The present case illustrates several important aspects of a ruptured GDAA. First, the diagnosis was challenging because the initial presentation was nonspecific, consisting primarily of diffuse abdominal pain and a single episode of coffee-ground emesis. Such symptoms may mimic a variety of gastrointestinal disorders, potentially delaying definitive diagnosis.

A notable feature of this case was the coexistence of chronic pancreatitis and severe atherosclerotic disease, both recognized risk factors for visceral artery aneurysm formation [2]. Chronic pancreatitis may contribute to vascular wall injury through persistent inflammation and enzymatic degradation of surrounding tissues, whereas atherosclerosis promotes structural weakening of the arterial wall [6]. The presence of both conditions may have increased the patient’s susceptibility to aneurysm development and rupture.

Although the lesion was diagnosed as a GDAA based on CTA and digital subtraction angiography, distinguishing a ruptured true aneurysm from a pseudoaneurysm may be challenging using imaging alone, particularly in patients with chronic pancreatitis. In the absence of histopathological confirmation, the diagnosis was based on the available radiological and angiographic findings.

Selection of the embolic agent is an important consideration in the endovascular management of visceral artery aneurysms. Available options include coils, liquid embolic agents, vascular plugs, and covered stents, with the choice depending on aneurysm morphology, vascular anatomy, collateral circulation, and operator experience. In the present case, coil embolization was selected because it allowed controlled and precise occlusion of the GDA.

The aneurysm rupture resulted in a surrounding hematoma, causing compression of the duodenum and gastric distension. This mass effect is an uncommon manifestation and contributed to the patient’s clinical presentation. Recognition of such indirect imaging findings may facilitate earlier diagnosis in similar cases.

Endovascular coil embolization provided rapid exclusion of the aneurysm from the circulation and avoided the need for open surgical intervention [7-8]. Although open surgical repair remains an important treatment option, particularly when endovascular therapy is not feasible or in cases of persistent hemorrhage, it is associated with greater procedural invasiveness, increased blood loss, and longer hospitalization. A recent systematic review and meta-analysis demonstrated that endovascular repair is associated with lower perioperative morbidity, shorter hospital stay, and comparable technical success and overall mortality compared with open surgery. Notably, among patients presenting with ruptured visceral artery aneurysms, mortality was lower after endovascular repair than after open surgery (4.1% vs. 31%), further supporting the role of endovascular therapy as the preferred treatment strategy in appropriately selected patients [7-10].

This case emphasizes the importance of considering visceral artery aneurysm rupture in patients presenting with abdominal pain and signs of upper gastrointestinal bleeding, particularly in the setting of chronic pancreatitis. Prompt cross-sectional imaging followed by endovascular treatment can be lifesaving and may prevent the need for more invasive surgical management.

## Conclusions

GDAA is a rare but potentially fatal vascular condition that requires prompt diagnosis and treatment, particularly in cases of rupture. This case demonstrates the critical role of contrast-enhanced CT and angiography in establishing the diagnosis and guiding management. Endovascular techniques represent the first-line treatment in most cases of visceral artery aneurysms and require careful planning to ensure effective exclusion of the aneurysm while preserving collateral circulation. In the present case, coil embolization provided rapid and effective exclusion of the aneurysm from the circulation, resulting in a favorable clinical outcome and avoidance of open surgical intervention. Early recognition and timely endovascular treatment may significantly reduce morbidity and mortality associated with ruptured GDAAs. Given the rarity of this condition, standardized screening and follow-up protocols are lacking; therefore, diagnostic evaluation and therapeutic management should be individualized based on the patient's clinical presentation and anatomical characteristics.
